# Supplementary material for: Stacking Model‐Based Classifiers for Dealing With Multiple Sets of Noisy Labels
Source: Biom J. 2025 Mar 12;67(2):e70042. doi: 10.1002/bimj.70042 (PMC11898607; doi:10.1002/bimj.70042)
Supplement: Supplementary file 1 — Supporting Information [file BIMJ-67-e70042-s001.zip › density-based-ensemble-model-main/competitors_real_data/netlab-master/netlab-master/help/gmmactiv.htm]

Netlab Reference Manual gmmactiv


# gmmactiv

## Purpose

Computes the activations of a Gaussian mixture model.

## Synopsis

```
a = gmmactiv(mix, x)
```

## Description

This function computes the activations `a` (i.e. the
probability `p(x|j)` of the data conditioned on each component density)
for a Gaussian mixture model. For the PPCA model, each activation
is the conditional probability of `x` given that it is generated
by the component subspace.
The data structure `mix` defines the mixture model, while the matrix
`x` contains the data vectors. Each row of `x` represents a single
vector.

## See Also

`gmm`, `gmmpost`, `gmmprob`

---

**Pages:**
Index


---

Copyright (c) Ian T Nabney (1996-9)
